# Transcriptomic analysis of broiler chickens reveals metabolic adaptations to a reduced crude protein diet

**DOI:** 10.1016/j.psj.2025.104920

**Published:** 2025-02-17

**Authors:** Collins Amponsah Asiamah, Sara de las Heras-Saldana, Sosthene Musigwa, Sarbast K. Kheravii, Shu-Biao Wu

**Affiliations:** a School of Environmental and Rural Science, University of New England, Armidale, NSW 2351, Australia; b Animal Genetics and Breeding Unit, a joint venture of NSW Department of Primary Industries and University of New England, Armidale, NSW 2351 Australia

**Keywords:** Transcriptome, Liver, Crude protein, Differentially expressed genes

## Abstract

Protein is an essential component of poultry diets and directly influences growth performance, profitability, and the environment. While the relationship between animal performance and nutrient intake is modulated by gene expression, the transcriptomic mechanisms underlying broiler adaptation to varying dietary protein levels remain underexplored. This research investigated the liver transcriptomic response of broiler chickens fed a reduced crude protein (**RCP**) diet, aiming to identify differentially expressed genes (**DEGs**) and metabolic pathways that may underlie protein metabolism and growth. A total of 256 as-hatched Cobb 500 broilers were fed standard starter and grower diets. At the finisher stage (days 19-28), birds were randomly assigned to one of two treatments: normal crude protein (**NCP**) or RCP diets, with eight replicates per treatment and 16 birds per replicate. The performance results showed no significant difference in weight gain or feed intake between the groups, but the feed conversion ratio was significantly higher (*P* < 0.05) in RCP-fed birds. Notably, protein utilization efficiency was significantly higher (*P* < 0.01) in RCP-fed birds than in NCP-fed birds. Transcriptomic analysis of NCP vs RCP revealed 28 DEGs with 9 upregulated and 19 downregulated, including *ACSL6, ME1, IGFBP2, HSD3B1, HAO2, MYO1A*, and *PNPLA3*. Functional enrichment analysis revealed significant involvement of DEGs (*P* < 0.05) in the *PPAR* signaling pathway and a tendency toward enrichment in the metabolic pathway (*P* = 0.053). These findings suggest that metabolic adaptations, supported by the DEGs and increased protein utilization, likely enabled RCP-fed birds to perform comparably to those fed the NCP diet. These insights reveal potential transcriptomic markers for optimizing reduced-protein diets in broiler production, aligning the industry goals of balancing productivity with sustainability.

## Introduction

Broiler production is a vital sector of the poultry industry and contributes to global food security and economic sustainability (Sheppard, 2004; Marangoni et al., 2015). However, the environmental impact and cost of broiler feeds rich in crude protein (**CP**) pose significant challenges for sustainable production. Although effective for rapid growth and performance, high-CP diets are often linked to increased nitrogen excretion, contributing to greenhouse gas emissions, environmental contamination, and escalating feed costs (Castanheira and Freire, 2013; Belloir et al., 2017). Researchers have explored reduced CP (**RCP**) diets supplemented with amino acids (**AA**) to maintain broiler performance while mitigating these environmental impacts (Attia et al., 2020; Strifler et al., 2024).

An RCP diet balanced with all AA offers a promising approach for decreasing nitrogen excretion without compromising growth (Attia et al., 2020, 2022; Benahmed et al., 2023). However, focusing on only essential AA (**EAA**) without considering non-essential AA (**NEAA**) may compromise overall performance, nutrient utilization, and carcass traits due to imbalances in EAA and NEAA (Benahmed et al., 2023; Musigwa et al., 2024). The EAA:NEAA ratio, or the essential-to-true protein (**E:T**) ratio, has emerged as a key factor in designing effective low-CP diets. Studies indicate an optimal E:T ratio is necessary to ensure growth, protein synthesis, and efficient nutrient utilization in RCP-fed broilers (Maia et al., 2021; Musigwa et al., 2024). Despite the potential benefits of RCP diets, the metabolic adaptations that allow broilers to maintain performance under low-protein conditions are still not well understood, particularly at the transcriptomic level.

Recent advances in high-throughput technologies, such as RNA sequencing (**RNA-seq**), have been well integrated into poultry research. RNA-seq allows the study of transcriptomics, which refers to the complete set of RNA transcripts expressed in a cell, tissue, or organism at a given time or condition. This technique provides a broader view of gene expression patterns, leading to the identification of differentially expressed genes (**DEGs**) that may play crucial roles in biological processes, metabolic pathways, and responses to dietary changes (Khwatenge et al., 2020; Schäfer et al., 2023). In the liver, a key organ for metabolism (Zaefarian et al., 2019), dietary protein levels and AA supplementation are known to influence gene expression related to growth and metabolic pathways such as lipid metabolism, glycolysis, and gluconeogenesis (Schroyen et al., 2021; Ma et al., 2023). When protein levels are reduced in diets, chickens often exhibit metabolic adaptations, activating alternative pathways or shifting resources to maintain essential biological functions, including growth and immune responses (Hu et al., 2024; Sutton et al., 2024). For example, lower dietary protein can upregulate genes associated with lipid metabolism pathways, promoting the use of fats as energy sources to compensate for reduced protein-derived amino acid diets (Schroyen et al., 2021; Hu et al., 2024).

Given the limited research on the molecular mechanisms associated with restricted crude protein, we hypothesize that an optimal 0.64 E:T RCP in broiler diets could influence liver gene expression, revealing metabolic pathways affecting growth in broiler chickens. Therefore, RNA-seq was utilized in this study to investigate liver transcriptomic changes in response to an RCP diet in broilers, focusing on identifying DEGs and enriched biological pathways. This study aims to clarify the metabolic adaptations that enable broilers to thrive on RCP diets with an optimal 0.64 E:T ratio. These findings will contribute to the ongoing effort to optimize broiler diets for sustainability, balancing production performance with environmental and economic benefits.

## Materials and methods

### Ethics statement

The Animal Ethics Committee of the University of New England approved all the animal experimental protocols, with approval number ARA22-032.

### Experimental animals and diets

Baiada Hatchery, Tamworth, New South Wales, Australia, supplied 256-day-old Cobb 500 broiler chicks. The chicks were weighed and randomly distributed into 16 floor pens, each housing 16 birds. Birds were fed in three phases: a common starter crumbled diet from day (**d**) 0 to d 8, a common grower pelleted diet from d 9 to d 18, and treatment pelleted diets (finisher) from d 19 to d 28. The experimental design comprised two distinct dietary treatments, each replicated eight times and randomly allocated to the pens, i.e., a normal crude protein (**NCP**) diet used as a control and a reduced crude protein (RCP) diet (Table 1). All diets were formulated following the Cobb 500 specifications (Cobb500, 2018), except the RCP diet which required modifications to adjust the CP level to an optimal E:T ratio of 0.64. Details on the estimation of the E:T ratio and the analysis of nutrients in the present study are described in our previous studies (Musigwa et al., 2024), where the optimal E:T ratio is 0.60. However, as opposed to our previous study, tyrosine was added to EAA in the current study, which led to an optimal E:T ratio of 0.64. The ingredients, calculated, and analyzed nutrient contents of the experimental diets are provided in Table 1, Table 2. The lighting and temperature schedule followed the Cobb 500 management guidelines (Cobb500, 2018).

**Table 1:** Feed ingredients and calculated nutrient levels.

Ingredients, %	Starter	Grower	Finisher	
			RCP	NCP
Wheat	17.0	28.0	22.0	20.0
Barley	20.0	20.0	25.0	25.0
Soybean meal	27.0	26.0	7.80	15.8
Wheat Pollard	9.90	5.00	11.4	17.0
Corn	10.0	10.0	10.0	2.00
Sorghum	1.00	1.00	10.0	8.00
Canola ml solvent	4.00		0.50	0.50
Canola oil	4.80	3.56	3.74	5.25
Rice hulls	0.93	1.74	1.00	0.50
Na Bentonite	1.50	1.50	1.50	1.00
Carbohydrases^1^	0.005	0.005	0.005	0.005
Phytases^2^	0.01	0.01	0.01	0.01
K Carbonate			0.654	0.325
Limestone	1.36	1.29	1.26	1.26
Monocalcium phosphate	0.67	0.51	0.56	0.44
Salt	0.24	0.11	0.11	0.19
Na bicarbonate	0.02	0.22	0.44	0.32
TiO_2_			0.50	0.50
Vitamin premix^3^	0.07	0.07	0.07	0.07
Trace mineral premix^4^	0.10	0.10	0.10	0.10
Choline Cl 70 %	0.11	0.06	0.13	0.11
L-lysine HCl 78.4	0.33	0.31	0.70	0.44
DL-methionine	0.29	0.25	0.24	0.20
L-threonine	0.13	0.12	0.32	0.20
L-valine	0.10	0.08	0.29	0.15
L-isoleucine			0.23	0.08
L-leucine			0.22	0.02
L-phenylalanine			0.06	
L-cystine			0.11	0.08
L-arginine FB			0.44	0.16
L-Glycine			0.26	
Total	100	100	100	100
**Calculated nutrient levels, % or indicated units**				
AME, MJ/kg	12.69	12**.**88	13.17	13.18
NE, MJ/kg	9.90	10.00	10.40	10.40
CP (N × 6.25)	23.00	21.00	16.00	18.00
d Lysine	1.220	1.120	0.995	0.995
d Cystine	0.311	0.297	0.328	0.328
d Methionine	0.569	0.509	0.418	0.418
d Threonine	0.817	0.750	0.697	0.697
d Arginine	1.280	1.180	1.075	1.075
d Phenylalanine	0.895	0.847	0.597	0.685
d Leucine	1.372	1.290	1.085	1.085
d Isoleucine	0.820	0.766	0.678	0.678
d Valine	0.939	0.860	0.796	0.796
d Glycine	1.383	1.307	1.050	1.029
Calcium	0.880	0.800	0.760	0.760
Phosphorus	0.440	0.400	0.380	0.380
Sodium	0.160	0.160	0.220	0.220
E:T			0.640	0.640

AME, apparent metabolizable energy; NE, net energy; CP, crude protein; E:T, essential amino acids-to-true protein; RCP, reduced crude protein; NCP, normal crude protein.

1 Rovabio® Advance (xylanase, β-glucanase and arabinofuranosidase).

2 AXTRA ® PHY Gold 10T (Dupont Animal Nutrition) provided 500 FTU/kg.

3 Formulated to supply 5040 mg retinol, 17.5 mg cholecalciferol, 105 mg tocopheryl acetate, 4 mg menadione, 4 mg thiamine, 11 mg riboflavin, 77 mg niacin, 18 mg pantothenate, 7 mg pyridoxine, 0.35 mg biotin, 3.0 mg folate, 0.02 mg cyanocobalamin per kg of finished feed.

4 Formulated to supply 23 mg copper, 1.79 mg iodine, 57 mg iron, 171 mg manganese, 0.43 mg selenium, and 143 mg zinc per kg finished feed.

**Table 2:** Analyzed concentrations of nutrients in experimental diets.

Nutrients, mg/g or indicated units	Finisher	
	RCP	NCP
CP, % as is	15.07	17.74
NE, MJ/kg	11.12	10.63
AME, MJ/kg	14.43	14.20
AMEn, MJ/kg	13.78	13.45
Histidine	3.21	4.20
Serine	5.71	7.66
Arginine	10.57	10.44
Glycine	7.40	6.81
Aspartic acid	9.40	13.54
Glutamic acid	26.60	36.05
Threonine	6.90	7.55
Alanine	5.54	7.05
Proline	9.29	12.31
Lysine	9.47	11.52
Tyrosine	2.93	3.38
Methionine	3.71	3.56
Valine	8.60	9.25
Isoleucine	6.92	7.57
Leucine	11.68	12.67
Phenylalanine	6.41	8.08

AME, apparent metabolizable energy; AMEn, AME corrected of nitrogen; NE, net energy; CP, crude protein; RCP, reduced crude protein; NCP, normal crude protein.

### Laboratory analysis of diets for protein utilization

To calculate the nutrient utilization of the birds, samples from the experimental diets were subjected to dry matter (**DM**) analysis by oven drying at 105°C until a consistent weight was obtained (Galyean and May, 1980). Protein utilization efficiency (WG/CP intake) was calculated as follows:

CPintake(g/b/d)=measuredCP%*FI(DM/b)

WG/CPintake(g/g/b/d)=WG(g/b/d)÷CPintake(g/b/d)

### Performance and sample collection

Weight gain (**WG**) and feed intake (**FI**) of birds per pen were measured on days 19 and 28 to calculate the feed conversion ratio (**FCR**). On d 28, one bird from each pen was selected based on the average body weight and euthanized for liver tissue collection, resulting in eight birds per treatment. The ventral part of the right lobe of the liver tissue was sampled immediately (1 cm^3^), washed briefly with sterile phosphate-buffered saline (**PBS**), placed in RNAlater, and kept at 4°C for 4 hr before being stored at -20°C for RNA extraction.

### RNA extraction and sequencing

RNA was extracted from sixteen liver tissues (8 birds/treatment) using Isolate II RNA Mini Kit (Bioline Meridian Bioscience, Australia) following the manufacturer's instructions. The RNA concentration and purity were measured with a NanoDrop ND-8000 spectrophotometer (Thermo Fisher Scientific, Waltham, MA), and RNA integrity was assessed with the Agilent 2100 Bioanalyzer using RNA 6000 Nano Kit (Agilent Technologies, Inc., Waldron, Germany). RNA samples with A260/230 values >1.8, A260/280 values ranging from 1.8 to 2.0, and RNA integrity numbers (**RIN**) > 7.5 were considered high quality. In this study, the RIN of the samples ranged from 8.5 to 10. The RNA samples were processed and sequenced by the Australian Genomic Research Facilities (Melbourne, Vic., Australia). Image analysis was performed in real-time by the NovaSeq Control Software (**NCS**) v1.7.5 and Real-Time Analysis (**RTA**) v3.4.4, running on the instrument computer. RTA performs real-time base calling on the NovaSeq instrument computer. Then the Illumina DRAGEN BCL Convert 07.021.624.3.10.8 pipeline was used to generate the sequence data.

### Transcriptome mapping and assembly

The quality of the raw reads was assessed with FastQC v.0.12.0 (https://www.bioinformatics.babraham.ac.uk/projects/fastqc/) (Andrews, 2010) and filtered with Trimmomatic v.0.39 software (Bolger et al., 2014). The reads that contained adapters or > 10 % of unknown nucleotides and low-quality bases (Phred score *Q* < 30) were filtered out to generate high-quality reads. Cleaned reads were mapped to the *Gallus gallus* reference genome (GRCg7b) using STAR v.2.7.9 software (Dobin et al., 2013). The mapped reads were assembled using HTSeq v.2.0.3 (Putri et al., 2022), which produced a gene count matrix.

### Differential expression, pathway, and gene ontology

For the differential expression analysis, a quality control assessment was performed on the gene counts, such as filtering of genes with very low expression levels followed by the normalization of the expression using the filterByExpr and calcNormFactors based on the trimmed mean of M-values method in the edgeR package v.4.0.16 (Robinson and Oshlack, 2010). A principal component analysis was performed to visualize the expression profiles and assess any technical variations or outliers among the samples that could influence the downstream analysis. Samples distinctly isolated from their respective group members exhibiting the lowest gene count within the group were identified as outliers and subsequently removed from further analysis. To identify DEGs between NCP and RCP, a generalized linear model was fit to the data using the glmQLFit function of the edgeR package v.4.0.16 (Robinson et al., 2010), which accounts for variability in gene expression and tests for differential expression between groups. Transcripts with an adjusted *P* < 0.05 and an absolute |log2 (fold-change)| ≥ 1 were considered DEGs. DEGs were subjected to Kyoto Encyclopedia of Genes and Genomes pathway (**KEGG**) and Gene Ontology (**GO**) analyses using the Database for Annotation, Visualization and Integrated Discovery (**DAVID** v.6.8) (Huang et al., 2009) to identify the enriched biological pathways and functional categories associated with these DEGs. GO enrichment analyses included biological processes, cellular components, and molecular functions, using a significance threshold of *P* < 0.05 and gene counts ≥ 2. All enrichment analyses used annotations specific to the *Gallus gallus* reference genome.

### Statistical analysis

The normality of the performance data (WG, FI, FCR, and protein efficiency) was assessed via the Shapiro-Wilk normality test. Subsequently, a student t-test was conducted to examine the impact of the treatments on the birds. Significance was set at a threshold of *P* < 0.05. All analyses were performed with R software v.4.1.2 (Team, 2021).

## Results

### Broiler performance and protein utilization

The effects of dietary treatment on broiler chickens' performance and protein utilization efficiency are shown in Table 3. Compared with the birds in the control group, those fed the RCP diet had significantly greater FCR and protein utilization (*P* < 0.05) from d 19 to 28. There was no significant difference in WG and FI between the two groups (Table 3).

**Table 3:** Effects of dietary treatment on broiler performance and nutrient utilization.

Treatment^1^	Mean^2^ (d 19-28)			
	WG, g/b/d	FI, g/b/d	FCR	WG/CP intake, g/g/b/d
RCP	100.2	161.6	1.614	4.599
NCP	104.0	158.8	1.527	4.137
Pooled SEM	1.232	1.540	0.016	0.067
*P-value*	0.128	0.392	0.0024	<0.0001

1 RCP, reduced crude protein; NCP, normal crude protein; SEM, standard error of mean.

2 WG, weight gain; FI, feed intake; FCR, feed conversion ratio; CP, crude protein.

### RNA-seq data analysis

**Fig. 1:**
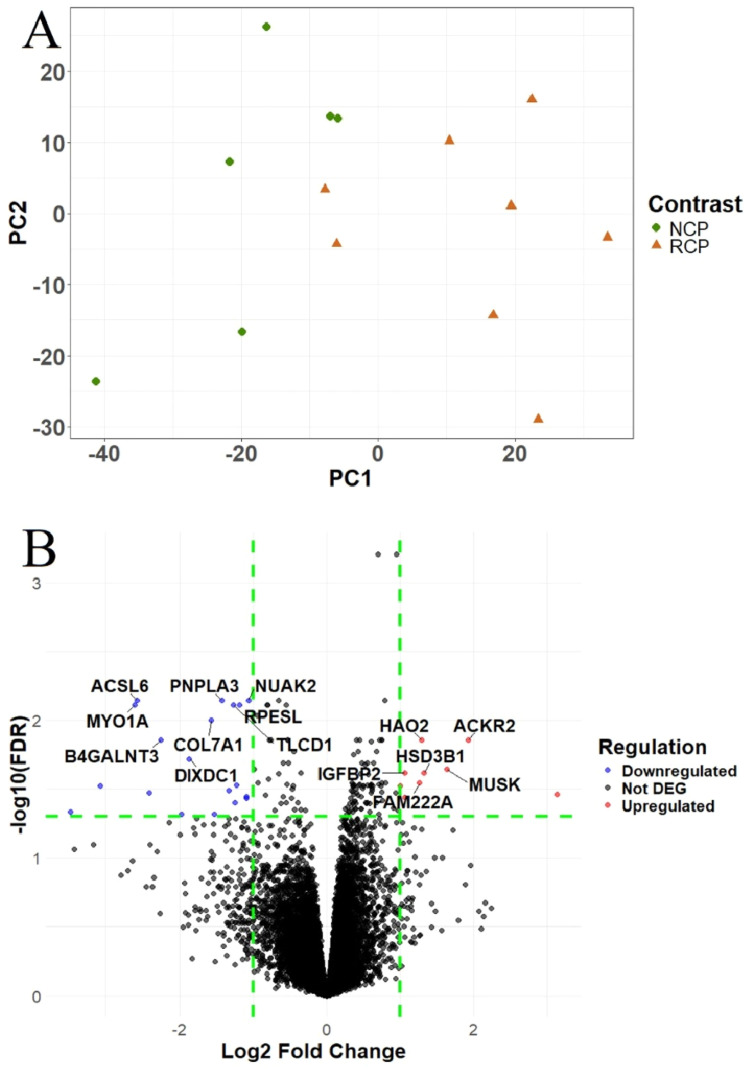
Differentially expressed genes in the liver of broiler chickens. A. Principal component analysis of samples. B. Volcano plot of DEGs in NCP vs RCP. The X-axis shows log2 fold change of DEGs between the two groups. The Y-axis indicates the –log10 (FDR) of gene expression variations. Each dot in the volcano plot represents a gene; red dots represent upregulated DEGs, blue dots represent downregulated DEGs and black dots represent the non-significant genes. RCP- reduced crude protein; NCP- normal crude protein.

The mean raw reads, clean reads, and clean pairs with a Phred score ≥ Q30 (%) for each treatment are shown in Table 4. The GC content of the samples ranged from 47 % to 50 %. Principal component analysis of the normalized gene counts revealed outliers, which were subsequently removed from further analysis (Fig. 1A).

**Table 4:** Summary of the number of reads obtained from the RNA sequencing.

Treatments^1^	Mean raw reads	Mean clean reads	Mean clean pairs
RCP	81,426,641	80,634,197	53,583,999
NCP	80,279,420	79,534,829	53,468,086

1 RCP, reduced crude protein; NCP, normal crude protein.

### Identification of differentially expressed genes

In this study, differential expression analysis was performed to detect differences in gene expression profiles between the two groups using cut-off values of |log2(fold change)| ≥ 1 and an adjusted false discovery rate (**FDR**) < 0.05 (Fig. 1B). In contrast NCP vs RCP, 28 DEGs were identified, of which 9 were upregulated and 19 were downregulated (Supplementary Table 1, Fig. 1B). The top 12 upregulated and downregulated DEGs with their functions are shown in Table 5.

**Table 5:** Functions of the top 12 upregulated and downregulated DEGs.

Gene names	logFC^1^	FDR^2^	Direction^3^	Functions	References
*HAO2*	1.294	1.39E-02	Upregulated	fatty acid metabolism in rats and beta-oxidation in laying hens	(Gloux et al., 2019; Zuo et al., 2024)
*ACKR2*	1.924	1.39E-02	Upregulated	decoy and scavenger for most inflammatory chemokines in humans	(Bonecchi and Graham, 2016)
*MUSK*	1.634	2.27E-02	Upregulated	agrin-induced formation of the neuromuscular junction in chickens	(Ip et al., 2000)
*IGFBP2*	1.066	2.41E-02	Upregulated	lipogenesis, and associated with growth and fatness traits in chickens	(Li et al., 2006; Leng et al., 2009; Mu et al., 2019)
*HSD3B1*	1.321	2.41E-02	Upregulated	lipid metabolic pathways in chickens	(Jing et al., 2022)
*FAM222A*	1.264	2.86E-02	Upregulated	increases amyloid beta aggregation in humans	(Alabdulraheem and Durdagi, 2023)
*ACSL6*	−2.575	7.19E-03	Downregulated	fatty acid activation and transport in chickens and muscular decosahexaenoic acid enrichment and lipid synthesis in fish	(Desert et al., 2018; Chen et al., 2022)
*NUAK2*	−1.061	7.19E-03	Downregulated	regulates cytoskeletal processes in humans	(Bonnard et al., 2020)
*PNPLA3*	−1.418	7.19E-03	Downregulated	involved in lipid metabolism in chickens	(Claire D'Andre et al., 2013)
*RPESL*	−1.186	7.76E-03	Downregulated	regulator of lipid transport in the retina in humans	(Simó et al., 2010)
*TLCD1*	−1.269	7.76E-03	Downregulated	regulates cellular phosphatidylethanolamine composition in mice	(Petkevicius et al., 2022)
*MYO1A*	−2.607	7.76E-03	Downregulated	involved in border brush, powering the release of vesicles from the tips of microvilli into the intestinal lumen in mice	(McConnell and Tyska, 2007; McConnell et al., 2009)

1 logFC, log fold change.

2 FDR, false discovery rate.

3 Direction: Indicates whether the gene is upregulated or downregulated. Upregulated genes are more highly expressed in birds fed the NCP diet than in RCP-fed birds, while downregulated genes are expressed at lower levels in NCP-fed birds compared to those fed the RCP diet.

### Gene ontology and KEGG pathway

A functional enrichment analysis was conducted to further investigate the functions of the DEGs. Only one of the four GO terms was significantly (*P* < 0.05) enriched (Table 6). The study revealed two main pathways: a significantly (*P* < 0.05) enriched *PPAR* signaling pathway and metabolic pathways showing a tendency (*P* = 0.053) (Table 6).

**Table 6:** Gene ontology and KEGG pathway analysis of DEGs.

Category	Term	Count	P-value	Genes
Biological Process	GO:0060070∼canonical Wnt signaling pathway	2	0.099	*WNT6, DIXDC1*
Cellular Component	GO:0005886∼plasma membrane	8	0.088	*RASGRF2, MYO1A, MUSK, KITLG, GPR142, TLCD1, DIO3, SLCO2B1*
	GO:0005615∼extracellular space	5	0.088	*COL7A1, TSKU, WNT6, SLCO2B1, IGFBP2*
Molecular function	GO:0005109∼frizzled binding	2	0.042	*WNT6, DIXDC1*
KEGG Pathway	gga03320: PPAR signaling pathway	3	0.008	*ACKR2, ACSL6, ME1*
	gga01100: Metabolic pathways	7	0.053	*ACKR2, HSD3B1, PNPLA3, B4GALNT3, HAO2, ACSL6, ME1*

## Discussion

Reduced CP diets supplemented with AA have been investigated to reduce feed costs and environmental footprints, but the compromise of bird performance by such diets halts the significant reduction in protein in the broiler feed to achieve these goals (Barekatain et al., 2023; Strifler et al., 2023). A nutritionally balanced, reduced-protein diet is essential to ensure both environmental benefits and cost reductions. On the other hand, understanding the metabolic pathways involved in the response of birds to these diets is critical for determining their biological implications, yet studies in this area are rare. Therefore, this study focused on assessing the effects of RCP diet on broiler performance and the liver transcriptomic profile.

In this study, both WG and FI of birds fed RCP diet were similar to those fed the control, i.e., NCP diet, except for FCR which was significantly higher in RCP-fed birds. Studies in Ross 308 broilers also reported no significant difference in WG and FI but inconsistent FCR values when CP levels were reduced by 1-3 % across a range of finisher-stage ages (20-42 days). Whereas Belloir et al. (2017) and Barekatain et al. (2023) reported a high FCR similar to the observed pattern in this study, Van Harn et al. (2019) and Lambert et al. (2023) described a lower FCR and Such et al. (2021) and Benahmed et al. (2023) reported no differences in FCR with low CP levels. Contrarily, in Arbor Acre broilers, even a modest 0.5-1 % reduction in CP resulted in significantly lower daily WG and increased FCR at the 22-42 days finisher stage (Qiu et al., 2023a). This discrepancy highlights the potential influence of broiler strains on growth performance and feed efficiency, as genetic differences can affect how birds metabolize protein and AA. Another plausible explanation for the variations in FCR outcomes with low CP diets lies in the dietary ingredients used. While this study utilized a wheat/barley/soybean meal-based diet, Benahmed et al. (2023) and Such et al. (2021), who observed no difference in FCR used a corn-soybean meal-based diet (Benahmed et al., 2023). It was established that maize-based diets are more conducive to CP reductions than wheat-based diets, likely due to the differences in starch composition, digestibility, and anti-nutritional factors (Chrystal et al., 2021; Selle et al., 2022). Other possible contributors to the divergent results could be the variations in bird age, AA balance, and even environmental conditions which were not reported.

The optimized E:T ratio in this study maximized WG, FI, and protein utilization efficiency (**WG/CP** intake) in RCP-fed birds. It is worth knowing that better protein utilization indicates less nitrogen (N) excretion as shown in earlier studies which reported reduced N excretion and improved N utilization with decreasing CP content (Lemme et al., 2019; Such et al., 2021). Literature suggests that formulating low CP diets focused on only EAA, without accounting for NEAA, may lead to an E:T imbalance, one of the factors contributing to poor performance (Hofmann et al., 2020; Camiré et al., 2023). Recently, Musigwa et al. (2024) recommended that an E:T ratio of 0.60 (without including tyrosine in EAA) in reduced protein diets is needed to maximize nutrient utilization, promoting growth similar to standard protein-fed birds. Other earlier studies have emphasized that an optimal E:T ratio is necessary for protein utilization and growth (Peres and Oliva-Teles, 2006). This aligns with findings in the current study, which utilized an optimized E:T ratio of 0.64 (with tyrosine added to EAA), emphasizing that the optimal E:T ratio in low CP diets enhances performance in terms of WG, FI, and protein utilization (Peres and Oliva-Teles, 2006; Musigwa et al., 2024). Despite an increased FCR in this study, adjusting E:T ratio to 0.64 contributed to comparable WG and FI, highlighting the importance of an optimal E:T ratio in low-protein diets.

Reduced CP diets and AA supplementation can modulate liver gene expression associated with growth and metabolic adaptation. In this study, DEGs such as *ACSL6, PNPLA3*, and *ME1* were upregulated in the liver of RCP-fed birds, suggesting potential shifts towards lipid metabolism as an adaptive energy source. Acyl-CoA synthetase long-chain family member 6 (***ACSL6*)** is integral in chicken hepatic fatty acid activation and transport, which are essential steps in lipid metabolism and energy production (Desert et al., 2018). In fish and mammals, *ACSL6* has been implicated in enriching docosahexaenoic acid and lipid synthesis in the brain, skeletal muscle, and other tissues (Teodoro et al., 2017; Chen et al., 2022). Malic enzyme 1 (***ME1***) is involved in lipid metabolism by facilitating the production of NADPH for hepatic fatty acid and cholesterol biosynthesis (Guay et al., 2007; Simmen et al., 2023). According to a transcriptomic study, *ME1* is involved in de novo fatty acid synthesis in the chicken liver, adapting to changes in dietary energy sources (Desert et al., 2018). *ME1* was reported to be upregulated in the liver and hypothalamus of fast-growing chickens compared with slow-growing chickens (Claire D'Andre et al., 2013). Studies have revealed that malic enzyme activity and mRNA expression were significantly higher in birds fed low-protein diets but lower in high-protein or basal diet-fed birds (Adams and Davis, 2001). Patatin-like phospholipase domain-containing 3 (***PNPLA3***), which was also upregulated in RCP-fed birds in this study, was associated with growth and carcass traits in F_1_ generation produced from Gushi chickens and Anka broilers (Su et al., 2012). *PNPLA3* involved in lipid metabolism was upregulated in the liver of fast-growing White Recessive Rock chickens (Claire D'Andre et al., 2013). These findings collectively suggest that the upregulation of *ACSL6, ME1*, and *PNPLA3* enhances energy efficiency and lipid utilization, likely compensating for the protein reduction in RCP diets and contributing to the observed protein efficiency and growth performance.

The upregulation of hydroxy‑delta-5-steroid dehydrogenase, 3 beta- and steroid delta-isomerase 1(***HSD3B1***) and hydroxyacid oxidase 2 (***HAO2***) in NCP-fed birds in this study may be involved in lipid metabolic pathways, carbon metabolism, and lactate oxidation in chickens (Zhang et al., 2021; Jing et al., 2022). A study in lean and fat broilers revealed that *HSD3B1* significantly increased in fat broilers more than in lean broilers (Jing et al., 2022), which aligns with the role of lipid metabolism in NCP-fed birds.

The enrichment of the *peroxisome proliferator-activated receptor* (***PPAR***) signaling pathway in this study further supports that RCP-fed birds adapt to lipid metabolism to enhance growth. The *PPAR* signaling pathway plays a critical role in systemic cell metabolism, energy homeostasis, and immune response regulation (Shi et al., 2024). *PPARs* are ligand-activated transcription factors within the nuclear hormone receptor superfamily that regulate metabolic networks by modulating lipid metabolism, glucose homeostasis, and energy balance (Christofides et al., 2021; Hassan et al., 2021). Additionally, *PPARs* enhance insulin sensitivity during adipocyte differentiation (Qiu et al., 2023b) and contribute to the control of inflammation and prevention of metabolic disorders such as atherosclerosis (Wagner and Wagner, 2020), highlighting their broad physiological importance. A comparative study of hybrid Ningdu Yellow chickens and their parental lines revealed that the enriched *PPAR* signaling pathway was involved in growth and development (Wu et al., 2024). These findings support the speculation that the enriched *PPAR* signaling pathway in the present study contributed to increased growth in RCP-fed birds. Moreover, the upregulation of DEGs including *ACSL6* and *ME1* in the *PPAR* signaling pathway of RCP-fed birds in this study further supports the idea that broilers can redirect energy pathways to maintain growth, potentially leveraging lipid metabolism pathways as adaptive responses to protein scarcity (Desert et al., 2018; Wu et al., 2024). Metabolic pathways, comprising a series of interconnected biochemical reactions, are critical for converting molecules into specific products while maintaining energy balance and cellular function. These pathways enable cells to obtain energy, synthesize macromolecules, and maintain homeostasis (Schuster et al., 2000). Several broiler studies have demonstrated that enriched metabolic pathways are involved in growth and development (Shao et al., 2022; Wu et al., 2024). It could be inferred that the potential enriched metabolic pathways with DEGs such as *ACKR2, HSD3B1, PNPLA3, B4GALNT3, HAO2, ACSL6*, and *ME1* identified in the present study suggest a shift toward lipid metabolism owing to the functions of most of the DEGs described above to enhance growth in RCP-fed birds. These metabolic pathways and adaptations likely enable birds to maintain body weight by utilizing alternative energy sources, such as fats, underscoring the adaptive potential of lipid pathways when dietary protein is reduced.

## Conclusion

This study demonstrated that broilers fed optimal 0.64 E:T RCP diets exhibit metabolic adaptations that enable comparable performance to those on normal protein diet. The upregulation of key DEGs, particularly those associated with *PPAR* signaling and metabolic pathways, underscores the ability of birds to adjust to reduced protein levels. These findings provide valuable insights into the potential of RCP diets to sustain growth, contributing to the broader goal of sustainable broiler production. Future research is needed to validate these results across diverse contexts and explore the practical application of these DEGs as markers for optimizing RCP diets in commercial broiler production.

## Disclosures

The authors declare that they have no competing interests.

## Appendix Supplementary materials

Image, application 1
