# Supplementary material for: Transcriptomic analysis of broiler chickens reveals metabolic adaptations to a reduced crude protein diet
Source: Poult Sci. 2025 Feb 17;104(4):104920. doi: 10.1016/j.psj.2025.104920 (PMC11889961; doi:10.1016/j.psj.2025.104920)
Supplement: Supplementary file 1 [file mmc1.docx]

**Supplementary Table 1. Differentially expressed genes in NCP vs RCP**

| Gene names | logFC^1^ | FDR^2^ | Direction^3^ |
| --- | --- | --- | --- |
| *HAO2* | 1.294087 | 0.013915 | Upregulated |
| *ACKR2* | 1.924286 | 0.013934 | Upregulated |
| *MUSK* | 1.634215 | 0.022745 | Upregulated |
| *IGFBP2* | 1.066085 | 0.024131 | Upregulated |
| *HSD3B1* | 1.321097 | 0.024131 | Upregulated |
| *FAM222A* | 1.264211 | 0.028627 | Upregulated |
| *TSKU* | 1.004367 | 0.030116 | Upregulated |
| *DIO3* | 3.138365 | 0.034393 | Upregulated |
| *SLCO2B1* | 1.067938 | 0.036335 | Upregulated |
| *ACSL6* | -2.57484 | 0.007192 | Downregulated |
| *NUAK2* | -1.06115 | 0.007192 | Downregulated |
| *PNPLA3* | -1.41798 | 0.007192 | Downregulated |
| *RPESL* | -1.18578 | 0.007757 | Downregulated |
| *TLCD1* | -1.2694 | 0.007757 | Downregulated |
| *MYO1A* | -2.60702 | 0.007757 | Downregulated |
| *COL7A1* | -1.56621 | 0.009981 | Downregulated |
| *B4GALNT3* | -2.25805 | 0.013915 | Downregulated |
| *DIXDC1* | -1.86507 | 0.019256 | Downregulated |
| *RASGRF2* | -1.22084 | 0.02974 | Downregulated |
| *RFLNA* | -3.08109 | 0.030116 | Downregulated |
| *GPR142* | -1.32702 | 0.032356 | Downregulated |
| *TMEM154* | -2.41584 | 0.033635 | Downregulated |
| *KITLG* | -1.0911 | 0.035614 | Downregulated |
| *CCNG2* | -1.09358 | 0.036909 | Downregulated |
| *ME1* | -1.24692 | 0.039877 | Downregulated |
| *WNT6* | -3.48013 | 0.046472 | Downregulated |
| *MXRA7* | -1.97678 | 0.048317 | Downregulated |
| *MRC2* | -1.52174 | 0.048317 | Downregulated |

**^1^** logFC, log fold change

^2^ FDR, false discovery rate

^3^Direction: Indicates whether the gene is upregulated or downregulated. Upregulated genes are more highly expressed in birds fed the NCP diet than in RCP-fed birds, while downregulated genes are expressed at lower levels in NCP-fed birds compared to those fed the RCP diet
